# Electrophysiology of subject-verb agreement mediated by speakers’ gender

**DOI:** 10.3389/fpsyg.2015.01396

**Published:** 2015-09-15

**Authors:** Adriana Hanulíková, Manuel Carreiras

**Affiliations:** ^1^Department of German Linguistics, University of FreiburgFreiburg, Germany; ^2^BCBL – Basque Center on Cognition, Brain and LanguageDonostia-San Sebastián, Spain; ^3^IKERBASQUE, Basque Foundation for ScienceBilbao, Spain; ^4^Departamento de Lengua Vasca y Comunicación, University of the Basque CountryDonostia, Spain

**Keywords:** subject–verb agreement, speaker’s gender, social language processing, speaker identity, ERP, P600, N400

## Abstract

An important property of speech is that it explicitly conveys features of a speaker’s identity such as age or gender. This event-related potential (ERP) study examined the effects of social information provided by a speaker’s gender, i.e., the conceptual representation of gender, on subject–verb agreement. Despite numerous studies on agreement, little is known about syntactic computations generated by speaker characteristics extracted from the acoustic signal. Slovak is well suited to investigate this issue because it is a morphologically rich language in which agreement involves features for number, case, and gender. Grammaticality of a sentence can be evaluated by checking a speaker’s gender as conveyed by his/her voice. We examined how conceptual information about speaker gender, which is not syntactic but rather social and pragmatic in nature, is interpreted for the computation of agreement patterns. ERP responses to verbs disagreeing with the speaker’s gender (e.g., a sentence including a masculine verbal inflection spoken by a female person ‘the neighbors were upset because I ^∗^stole_MASC_ plums’) elicited a larger early posterior negativity compared to correct sentences. When the agreement was purely syntactic and did not depend on the speaker’s gender, a disagreement between a formally marked subject and the verb inflection (e.g., the woman_FEM_^∗^stole_MASC_ plums) resulted in a larger P600 preceded by a larger anterior negativity compared to the control sentences. This result is in line with proposals according to which the recruitment of non-syntactic information such as the gender of the speaker results in N400-like effects, while formally marked syntactic features lead to structural integration as reflected in a LAN/P600 complex.

## Introduction

An important aspect of language comprehension is that listeners are able to efficiently establish the relation between words in an utterance and to extract meaning in just the right way. In order to capture syntactic dependencies between words and their features, listeners have to keep track of surface-level agreement between the form of one linguistic unit such as the noun *cat* and another unit such as the verb *scratches*. In English, the utterance *the cat scratches* reflects a standard use of number feature agreement between the subject and the verb whereas the sentence *the cat scratch* does not. Examining the way in which listeners respond to the standard use of agreement features provides insight into how relations between words are computed and how computational problems related to the rules of a given language or a variety are solved. Although languages vary greatly in how they reflect dependencies between words (see e.g., Corbett, 1991), previous research has shown that these dependencies are utilized and facilitate language processing in general (e.g., MacWhinney et al., 1984; Boelte and Connine, 2004; for a review see Friederici and Jacobsen, 1999).

Successful comprehension also entails the encoding of extra-linguistic information such as speaker-related characteristics. The processing of the non-standard utterance *the cat scratch* produced by a small child may not be hindered if a listener is able to use extra-linguistic information about the child’s incomplete mastery of the standard verb singular agreement. In such a case, anticipating a non-standard use of syntactic dependencies could potentially facilitate rather than hinder the overall processing effort (e.g., Hanulíková et al., 2012). Anticipations can result from a listener’s experience with certain speakers and their language use and can help in the interpretation and prediction of upcoming events across various modalities (e.g., Bar, 2007; Lau et al., 2014). But do listeners use speaker-specific characteristics for the computation of grammatical agreement features? Despite numerous studies on agreement, little is known about syntactic computations generated by speaker characteristics extracted from spoken language.

The question regarding how linguistic processing and speaker characteristics interact in real time has increasingly attracted research interest in cognitive neuroscience; in particular questions such as which neural mechanisms are involved and what is the time-course of speaker integration (e.g., Lattner and Friederici, 2003; Van Berkum et al., 2008; Scharinger et al., 2011; Hanulíková et al., 2012; Bornkessel-Schlesewsky et al., 2013). Such research suggests that speaker-related attributes such as gender, social group affiliation or age modulate speech perception, lexico-semantic processing and the processing of stereotypical knowledge, albeit the exact time-course varies across different linguistic levels. In contrast, little is known about how social information conveyed by a speaker’s voice affects syntactic processing (e.g., Hanulíková et al., 2012).

In many languages, syntactic relations between words usually include grammatical features such as person, number and gender but also features that go beyond the sentence given and include pragmatic aspects such as features of the speaker or the addressee. Slovak (a West-Slavonic language), for example, has a rich agreement paradigm that marks multiple properties simultaneously (Corbett, 1991), involving features for number, case, and gender. Each Slovak noun bears one of three grammatical genders (masculine, feminine, or neuter) and requires agreement with determiners, attributives, predicate adjectives, verb participles, and – in the past tense – with finite verbs (Badecker and Kuminiak, 2007, p. 82). The past tense is formed with the auxiliary “be” and the so-called *l*-participle (used as active but not as passive participle) that agrees with the subject of the clause in number and gender (e.g., Migdalski, 2006). In the third person no auxiliary is present, the past tense is expressed by the *l*-participle alone. It is called *l*-participle to reflect the fact that *l* is present in all suffixes of the participle (-*l* for masculine singular, -*la* for feminine singular, -*lo* for neuter singular). For example, the Slovak female past verb form *išla* (‘went_FEM_’) in an utterance such as *ja som išla* (‘I went_FEM_’) agrees with the biological gender of the female speaker (the personal pronoun *ja* ‘I’ is unmarked for gender and is often omitted due to Slovak being a pro-drop language). The correctness of the verb can be evaluated based on the conceptual (i.e., biological) gender of the speaker as conveyed by the speaker’s voice. The utterance *ja som išla* produced by a male voice would clearly be considered ungrammatical due to the mismatch between the female participle form (*išla*) and the speaker’s male gender.

Such speaker-related agreement features are found in many world languages. While within the Indo-European languages verb agreement with gender is common mainly in the Slavic subgroup (but partly also present in the French orthography), gender agreement on predicate adjectives can be seen in several languages (e.g., in Spanish *yo estaba cansada* ‘I was tired_FEM_’). Speaker-related gender agreement features can be considered pragmatic, because they are grammaticalized – encoded in the syntactic structure of a given language – and mark a relation between language and context (see Levinson, 1983, p. 9). Such pragmatic aspects (i.e., speaker-related physical/social information) of grammatical agreement processes have rarely been investigated. The exact nature of pragmatic (speaker-related) agreement processes compared to syntactic (speaker-independent) agreement processes remains unclear. In the present event-related potential (ERP) study, therefore, we examined pragmatic aspects of subject–verb-gender agreement by using electroencephalography (EEG) that allows for the examination of brain activity as speech unfolds over time without an additional interfering task (e.g., Hagoort and Brown, 2000; Van Berkum et al., 2008; Hanulíková et al., 2012). More specifically, we examined the nature and time course of the integration of a speaker’s voice during computation of grammatical gender agreement, and how such agreement processes compare to agreement computations that depend on the formal grammatical gender of the subject and are independent of a speaker’s voice.

### ERP Studies on Grammatical Gender Agreement

Communication in languages with a rich inflectional system requires comprehenders to keep track of agreement features between words. Numerous ERP-studies have demonstrated that the human brain shows distinct responses to expected as opposed to unexpected use of dependencies between words during sentence processing across many languages (for a review see Molinaro et al., 2011; Steinhauer and Drury, 2012). An important and well-studied grammatical category and agreement feature across languages is gender. Gender is usually considered an inherent feature of nouns and can be either assigned based on the meaning or the form of the noun or be an arbitrary formal feature (e.g., Corbett, 2006). Gender has been central to numerous studies examining how listeners store gender information in the brain and how it affects language production and comprehension in general and agreement processes in particular. It has been shown that listeners are sensitive to the correct use of gender, as is reflected in several studies showing that expected (congruent) gender is easier to process than unexpected (incongruent) gender (e.g., Grosjean et al., 1994; Bates et al., 1996; Boelte and Connine, 2004; for a review, see Friederici and Jacobsen, 1999). Most studies involving gender agreement effects examined grammatical gender agreement between nouns and determiners or nouns and adjectives (e.g., Münte and Heinze, 1994; Hagoort and Brown, 1999; Gunter et al., 2000; Barber and Carreiras, 2005; Van Berkum et al., 2005; Martin et al., 2012, 2014; see also Molinaro et al., 2011 for a review), while studies examining the processing of subject–verb agreement usually focus on features such as number (e.g., Kutas and Hillyard, 1983; Hagoort et al., 1993; Osterhout and Mobley, 1995; Kaan, 2002; De Vincenzi et al., 2003; Roehm et al., 2005; Silva-Pereyra and Carreiras, 2007; Zawiszewski et al., 2015) and person (e.g., Münte and Heinze, 1994; Hinojosa et al., 2003; Silva-Pereyra and Carreiras, 2007; Mancini et al., 2011; Shen et al., 2013; Zawiszewski et al., 2015). These studies have shown specific components responsive to incorrect agreement patterns such as an anterior negativity that is often left-lateralized (LAN) and peaks between 300 and 500 ms after the mismatch onset and/or a late posterior positivity (P600) peaking around 600 ms after the mismatch onset. While there still is ongoing debate about the functional significance of linguistically relevant ERP components, researchers frequently link LAN to an index of early syntactic processing (e.g., Friederici, 2002; Molinaro et al., 2011; Batterink and Neville, 2013) and a ‘failure to bind’ (Hagoort, 2003). The P600 on the other hand is typically associated with a later stage of processing and has been observed in response to various syntactic violations (e.g., Gouvea et al., 2010). It is assumed to index processes of syntactic integration, reanalysis, or recovery from well-formedness conflicts (Osterhout and Holcomb, 1992; Hagoort et al., 1993; Friederici, 1995, 2002; Bornkessel-Schlesewsky and Schlesewsky, 2009; for a review see Kutas and Federmeier, 2007) and may reflect controlled and strategic processes (Gunter et al., 1997; Coulson et al., 1998; Hahne and Friederici, 1999) or the competition between several syntactic unification alternatives (Hagoort, 2003).

Only a few prior studies have directly examined the processing of subject–verb agreement including grammatical gender features, i.e., cases in which verbs agree with the subject of the clause in gender. Deutsch and Bentin (2001) examined subject-predicate-gender agreement in Hebrew, in which the subject must agree with the predicate with regard to gender and number (or person for future, past, and imperative verb forms). ERP responses to predicates that were congruent or incongruent in gender with an animate (e.g., *boy*) or an inanimate (e.g., *diamond*) subject were recorded. Unlike many previous studies that report a P600 to subject–verb agreement, Deutsch and Bentin (2001) observed a larger modulation of the N400 to incongruent relative to congruent predicates, which was more pronounced in the animate than in the inanimate condition. Furthermore, an early left anterior negativity (eLAN) was observed but only in the singular animate condition. The eLAN is an early ERP component frequently linked to phrase structure violations (e.g., Friederici, 2002). The N400 is one of the most studied ERP components often seen during semantic processing. A consistent finding across studies on the N400 effect is that its amplitude is negatively correlated with the fit of a word in the (semantic) context. The N400 has frequently been interpreted as reflecting conceptual/semantic integration or a cognitive cost associated with word recognition, often linked to predictive processing (e.g., Kutas and Hillyard, 1980; Brown and Hagoort, 1993; Kutas and Van Petten, 1994; Federmeier, 2007). The N400 result for incongruent predicates in Deutsch and Bentin’s (2001) study was somewhat surprising and led to a discussion about the exact roles of formal gender and conceptual gender in agreement computations. Following Deutsch and Bentin (2001), it is the salient semantic information of an animate noun that usually functions as the thematic role of an agent and may lead to a more prominent N400 effect for the animate subject in contrast to the inanimate subject (see also Mancini et al., 2011 for similar N400-like effects in person mismatches in subject-verb agreement in Spanish).

Similar to Hebrew, Hindi future tense verbs agree in person, number, and gender with the subject of a sentence. In contrast to the Hebrew results, however, Nevins et al. (2007) observed a P600 to Hindi verb-gender agreement incongruencies relative to congruencies and no LAN or N400 effects. The discrepancy between the outcomes of the two studies could be explained by the fact that, while many languages use gender features, the extent to which gender information is used during syntactic processing may be language specific. Moreover, whether a LAN is observed may depend on specific linguistic properties, as well as on the methodology applied in a specific ERP study (for a critical discussion of the methodology used in the Hebrew study, see Molinaro et al., 2011; for a discussion of the LAN component, see Molinaro et al., 2014; Tanner, 2014). Taken together, the majority of syntactic agreement studies have observed that agreement violations lead to a P600 response or to a LAN followed by a P600. A similar pattern of results should be observed for Slovak subject–verb-gender incongruencies.

### Integration of Speaker Information in Language Processing

Phonetic and voice information are extracted from the speech signal early and in parallel (e.g., Knösche et al., 2002). Voice perception studies have shown that listeners automatically extract speaker-related information such as gender, age or estimates of body size (e.g., Mullennix et al., 1995; van Dommelen and Moxness, 1995; Braun and Cerrato, 1999; Cerrato et al., 2000). An important question is whether and when in time this speaker-related information is integrated during language processing. Word, sentence and discourse processing studies suggest that listeners anticipate what might be said and use their world knowledge or stereotype-driven inferences about a speaker during linguistic processing, but the exact timing of speaker integration differs across studies (e.g., Lattner and Friederici, 2003; Van Berkum et al., 2008; Scharinger et al., 2011; Hanulíková et al., 2012; Bornkessel-Schlesewsky et al., 2013).

Electroencephalography-studies show that conflicts with inferences about what a given speaker may say lead to qualitatively distinct ERPs (Van Berkum et al., 2008). Van Berkum et al. (2008) presented participants with utterances that were either consistent or inconsistent with a speakers’ age, gender, or socioeconomic status (e.g., the biologically implausible utterance produced in a male voice *I might be pregnant because I feel sick*). Van Berkum et al. (2008) found that inconsistencies between the speaker’s identity and the meaning of an utterance elicited a larger N400 compared to speaker consistency (e.g., hearing a woman producing the word *pregnant* in the above utterance). This modulation of the N400 effect suggests that listeners use speaker-related attributes in the earliest stages of meaning construction. In contrast to this finding, Lattner and Friederici (2003) suggest that the neural integration of speaker at the semantic level occurs relatively late. In their study, stereotype-driven inferences about a speaker in self-referent utterances such as *I like to wear lipstick* produced by a male speaker resulted in a P600 effect relative to the same utterance produced by a woman. Lattner and Friederici (2003) suggest that their result supports the idea that the P600 reflects a ‘re-integration of semantic meaning and stereotypical beliefs’ (Osterhout et al., 1997).

The distinct time-course patterns in these two studies could be attributed to the type of semantic/pragmatic context established by stereotypically driven inferences based on speaker characteristics. While Lattner and Friederici’s (2003) study measured the effect of speaker gender on sentence final stereotypical nouns (e.g., *lipstick, skirt, soccer*), Van Berkum et al.’s (2008) study was less restricted to the use of gender stereotypical role nouns and varied speakers’ gender, age, and accent (e.g., *I drink some wine before I go to sleep* in a child voice; *My favorite book is the fairy tale Sleeping Beauty* in an adult voice). Taken together, these studies suggest that violations of stereotypical role nouns as in Lattner and Friederici (2003) are likely to elicit a P600 (e.g., Osterhout et al., 1997), while the semantic-pragmatic incongruity as in Van Berkum et al. (2008) is more likely to elicit an N400 (e.g., Irmen et al., 2010). Since the pragmatic agreement examined in the present study relies on semantic-pragmatic congruity between the conceptual gender of the speaker and the predicate verb, it would be plausible to expect that pragmatic agreement involves the evaluation of speaker characteristics and reflects integration difficulties at the conceptual rather than purely syntactic level.

### The Present Study

The majority of studies on syntactic processing that employ grammatical agreement were conducted in the visual modality whereas studies that manipulate speaker characteristics in the domain of auditory processing usually do not examine syntactic processing (for a review, see Kutas and Federmeier, 2007). The present study fills this gap by examining agreement computations between verbs and a speaker’s gender in Slovak. We compared Slovak listeners’ ERP responses to Slovak past verb forms (a) agreeing or disagreeing with the conceptual gender of the speaker (first person singular; hence pragmatic agreement) and (b) agreeing or disagreeing with the grammatical gender of the animate subject (third person singular; hence syntactic agreement).

In line with previous research on gender agreement conflicts, we expected that incongruencies between the grammatical gender of an animate subject (e.g., *žena* ‘woman_FEM_’) and a predicate (e.g., *išiel* ‘went_MASC_’) would result in a P600 and possibly a LAN relative to the congruent predicate (e.g., *išla* ‘went_FEM_’). If the computation of the pragmatic agreement resembles the syntactic agreement, similar gender incongruency effects should be observed for the first and third person agreement features. There are, however, alternative accounts for the integration of speaker information during computations of verb agreement in the pragmatic condition. Following Nevins et al. (2007), the computation of concord might take place in a bottom-up fashion during the syntactic build-up of a sentence. Agreement processing starts once gender features are identified upon hearing a verb. This triggers a search for the subject (personal pronouns are, however, not marked for gender and are omitted due to Slovak being a pro-drop language) to check for matching gender features. This checking process may be independent of the semantic-pragmatic information (for a discussion, see Mancini et al., 2013) that is provided by a speaker’s voice. Under this assumption, pragmatic violations should not elicit any mismatch effects because the personal pronoun is unmarked for gender and no mismatch of the verb will be encountered (the verbal inflection is incorrect only if the pragmatic information about the speaker is considered and integrated in the syntactic build-up of the utterance).

Given prior research on the impact of speaker characteristics on linguistic processing, it seems unlikely to expect that pragmatic information is not used in the checking process. We therefore consider two possible outcomes. Following Nevins et al. (2007), it could be that the processing of agreement features that must be matched with the speaker’s gender can start before hearing the verb in a top–down fashion. Under this assumption, listeners would not wait until the presentation of the verb to initiate the agreement processing. Rather, listeners check whether the verb matches the speaker’s features that have been predictively built (Nevins et al., 2007). Since speaker information spreads across the entire utterance, listeners would quickly encounter a mismatch upon hearing the verb. If this mismatch is perceived as syntactic in nature, a P600 and possibly a LAN would be expected. Alternatively, in line with studies on the integration of speaker information, the mismatch could be perceived as pragmatically implausible. Since speaker characteristics convey social and pragmatic information whose violation have been shown to elicit an N400 effect (Van Berkum et al., 2008), it would be plausible to expect an N400 effect to violations of the pragmatic agreement. Such a result would also be in line with proposals according to which the recruitment of non-syntactic information about a person leads to conceptual/semantic integration reflected in an N400-like effect (e.g., Deutsch and Bentin, 2001; Mancini et al., 2013), while formally marked syntactic features lead to structural integration as reflected in a LAN/P600 complex (e.g., Molinaro et al., 2011).

## Materials and Methods

### Participants

Thirty-two native speakers of Slovak with no neurological or psychiatric disorders and no reported hearing problems volunteered to participate. They were all students (16 female, all right handed, mean age = 21, range = 18–24) at the Comenius University in Bratislava. All students grew up speaking Slovak only, and 27 of the students indicated communicative competence in at least one foreign language (the majority in English and German). Students received financial compensation for their participation; informed consent was obtained from all participants.

### Materials

The stimuli consisted of 240 sentences all of which contained a main clause followed by a subordinate clause. Each subordinate clause contained a past verb form that agreed in grammatical gender with the third person animate subject (e.g., *lebo svokra kradla* ‘because the mother-in-law_FEM_ stole_FEM_’) or with the conceptual gender of the speaker in the first person (*lebo som kradla* ‘because I stole_FEM_’). Each sentence was recorded in eight versions (see **Table 1** and the Supplementary Material for example sentences). A male speaker and a female speaker spoke a correct and an incorrect version of each sentence. The resulting 1920 sentences were distributed over eight experimental lists with one of the eight versions of each sentence occurring in only one experimental list. Within one experimental list, the number of correct and incorrect sentences was equally spread across conditions and voices. An additional set of four practice sentences with the same type of agreement patterns was recorded. The critical verbs at which the agreement violation became apparent were always embedded in a subordinate clause, at least two syllables before the end of the entire utterance. The critical verbs were between two to five syllables long. Verbs with the feminine inflections ended with the inflectional morpheme *-la* while the masculine inflections ended with *-l*. The mean logarithmic critical word form frequency per million was 0.76 (SD 0.76) for masculine verb forms and 0.43 (SD 0.76) for feminine verb forms (Slovenský Národný Korpus, 2009). Except for nine verbs (most of them with a stereotypically female connotation such as *to cook, to clean, to paint nails*), the masculine verb forms were more frequent that the feminine verb forms. This is not surprising because Slovak (and many other languages) use generic masculine nouns to refer to male beings, as well as to beings of unspecified sex (e.g., *pracovník* ‘worker_MASC_,’ *pracovníci* ‘workers_MASC_’), while the female nouns refer only to female beings (e.g., *pracovníčka* ‘worker_FEM_,’ *pracovníčky* ‘workers_FEM_’). This pattern of usage is then reflected in the frequency distribution of the inflected verb forms as well as nouns. All nouns in the subject position in the third person utterances were balanced for gender (half were male) and referred to professions or social groups (e.g., *translator, professor, teacher, member, tourist, friend*) or relatives (e.g., *mother-in-law, father, bride, brother, niece, cousin*). The grammatical gender of the subject always corresponded to the biological gender (neuter nouns such as *dievča* ‘girl’ were not used). The mean logarithmic word form frequency of the masculine nouns was 0.68 (SD 0.72) and of the feminine nouns -0.047 (SD 0.77) (Slovenský Národný Korpus, 2009).

**Table 1:** Sentences with subject–verb-gender agreement with English translation.

First person: pragmatic agreement (*n* = 60 correct and 60 incorrect)
*Female speaker: Susedia sa nahnevali, lebo som kradla/^∗^kradol slivky*
*Male speaker: Susedia sa nahnevali, lebo som ^∗^kradla/kradol slivky* (neighbors themselves upset because am stole_FEM_/stole_MASC_ plums) ‘neighbors were upset because I stole plums’
Third person: syntactic agreement (*n* = 60 correct and 60 incorrect)
*Female speaker: Susedia sa nahnevali, lebo svokrakradla/^∗^kradol slivky*
*Male speaker: Susedia sa nahnevali, lebo svokra kradla/^∗^kradol slivky* (neighbors themselves upset because mother-in-law_FEM_ stole_FEM_/stole_MASC_ Plums) ‘neighbors were upset because the mother-in-law stole plums’

*Critical words are underlined. Asterisk indicates an incorrect verbal inflection in a given context.*

Sentences were spoken by a 31 year-old male speaker and a 33 year-old female speaker. The speakers were siblings and grew up speaking a standard variety of Slovak. Their voices clearly indicated their biological gender as determined by ratings from 8 additional participants (mean age 28; 6 women), none of whom took part in the EEG study. On a scale from 1 to 5 (with 1 meaning clearly male voice and 5 meaning clearly female voice), the male speaker had an average of 1 and the female speaker had an average of 5. There was no ambiguity with respect to the gender of the speakers given their voice characteristics.

Both speakers received a complete list of all sentences, each sentence with its correct and incorrect version. They read the sentences at a natural speech rate. To minimize possible differences in the speech rate and intonation across the male and the female speakers, and across the conditions, each sentence was first produced by one speaker and immediately repeated by the second speaker (as in Hanulíková et al., 2012). Utterances that differed in prosody or speech rate were repeated by both speakers in both the correct and incorrect versions. Correct and incorrect versions of each utterance were produced in pairs to keep them as comparable as possible across conditions. In sentences spoken by the female speaker, the mean duration of the critical verbs was 474 ms (SD 104) and the mean duration of the whole sentence was 3578 ms (SD 725). The mean duration of the critical verbs spoken by the male speaker was 473 ms (SD 112) and the mean duration of the whole sentence was 3594 ms (SD 729). There were no significant differences in duration between the male and female speakers for either sentence duration or word duration (all *p*’s > 0.4). All sentences were adjusted in Praat to have comparable amplitude.

### Procedure

After the completion of an informed consent form, participants were seated in a comfortable armchair in front of a computer in a quiet room. They were told that they would listen to a male speaker and a female speaker talking about their lives. The 240 utterances were presented over loudspeakers situated next to the computer. Participants were asked to carefully listen for comprehension in order to answer comprehension questions that would follow some of the utterances. These questions (24 yes/no questions, half of which required a “yes” response) were included to ensure that participants were paying attention. To keep the task as natural as possible, and to keep the study comparable to previous task-less studies (e.g., Hagoort and Brown, 2000; Van Berkum et al., 2008; Hanulíková et al., 2012), no further grammaticality judgment or acceptability task was used. Participant performance of the comprehension questions was very high (mean percentage correct 98%, SD 4.46, range 83.3–100%). After the presentation of each utterance, a cross appeared in the middle of the screen to indicate that participants could blink or move. Participants were given button-press control over the initiation of the next trial, which started with a silence of 1000 ms followed by the utterance. The experiment consisted of six blocks and five short breaks. After the EEG study, participants were asked to complete the Edinburgh handedness test (to control for variation in lateralization of brain functions), a language-background questionnaire and comprehensibility ratings for the male and the female speakers. The ratings revealed that both speakers were equally well comprehensible. On a scale from 1 to 5 (with 1 meaning well comprehensible and 5 not comprehensible), both speakers had an average of 1.34.

### EEG Recording

Electroencephalography was recorded from 27 Ag/AgCl electrodes (impedance was kept below 5 kΩ) at standard locations (Fz, Cz, Pz, Fp1/2, F3/4, F7/8, FC1/2, FC5/6, C3/4, T7/8, CP1/2, CP5/6, P3/4, P7/8, O1/2). Two additional mastoid electrodes (placed on the left mastoid A1 and on the right mastoid A2) and four additional electrooculogram electrodes (placed above and below each eye) for eye movement and blink artifacts recordings were used. All recordings were referenced to the left mastoid during online recording, amplified with BrainAmp DC amplifiers (0.016–100 Hz band pass, digitized at 250 Hz), and re-referenced oﬄine to the mastoid average. EEG segments ranging from 200 ms before to 1200 after critical word onset were extracted and baseline corrected to a 200-ms pre-onset baseline. All segments with potentials above ±75 μV were rejected as artifacts (average segment loss 14%, range 13–15%, no differences between conditions). The segments were averaged per participant and condition, and mean amplitudes were analyzed with repeated-measures analyses of variance (ANOVAs). As a first step, the variation of effect size over all electrodes was examined, after which a topography-oriented analysis was conducted involving anterior (Fp1/2, F3/4, F7/8, FC1/2, FC5/6, Fz) and posterior distributions (CP5/6, CP1/2, P7/8, P3/4, O1/2, Pz).

For the statistical analyses, we followed the same analyses steps as in Hanulíková et al. (2012) and the time window was chosen in line with previous research (for an overview of EEG studies in the visual modality, see Molinaro et al., 2011) and on the basis of a visual inspection of the averaged data. For the P600 effect, the time window was 500–1000 ms (for a similar time-window, see e.g., Sassenhagen et al., 2014; for a short review, see Osterhout et al., 2012), for the LAN effect it was 200–500 ms (for a similar time-window, see e.g., Kutas and Hillyard, 1983; Roehm et al., 2005), and for the N400 effect it was 100–400 ms (for a similar time-window, see e.g., Ye et al., 2006; Shen et al., 2013). The ERP effects within the auditory modality might deviate somewhat from effects observed in the visual modality. Note that the detection of the agreement error is only possible once the critical verb has been heard, recognized, and the gender of the inflectional ending becomes available. Since the verbs varied in length between two to five syllables, we wanted to make sure that violation effects were captured correctly. The critical point within a verb to which the ERPs were time-locked was therefore set to onset of the last syllable that indicated the gender disambiguation (e.g., the onset of the syllable *dla* in the verbs *kradla* ‘stole_FEM_,’ *dohodla* ‘agreed_FEM_’ and the syllable *dol* for the verbs *kradol* ‘stole_MASC_,’ *dohodol* ‘agreed_MASC_’). Note that the disambiguation is possible already at the onset of the syllable (i.e., *d*) because phonetic properties of the onset of the critical syllables are affected by the following speech sounds. Similar time-locking procedures to the gender inflection of the critical word or to the ends of verb stems were applied in other auditory ERP studies (e.g., Van Berkum et al., 2005; Shen et al., 2013), resulting in somewhat early onsets of ERP effects.

## Results

### Speaker-Independent Agreement

**FIGURE 1:**
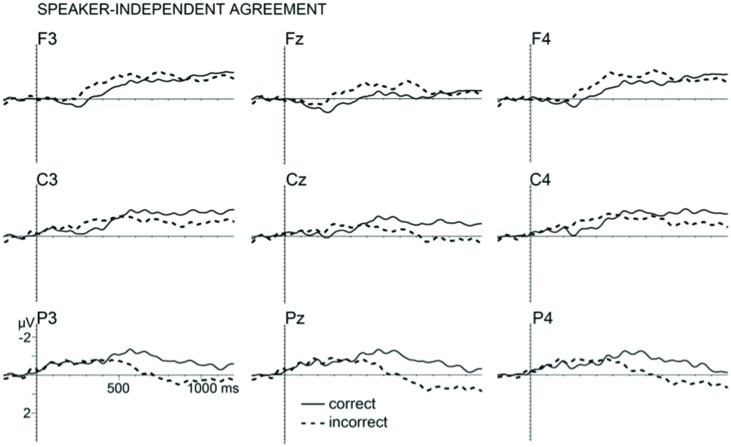
**Grand average event-related potentials (ERP’s) from nine electrodes elicited by third person incorrect verb agreement (dashed lines) and third person correct verb agreement (solid lines).** Waveforms are filtered (5 Hz high cutoff, 12 dB/oct) for presentation purpose only.

As can be seen in **Figure 1**, subject–verb-gender violations in the speaker-independent condition (syntactic agreement in the third person singular) resulted in a larger anterior negativity followed by a larger posterior positivity (P600) compared to correct utterances. The effect size varied over all electrodes as confirmed in a repeated measures ANOVA with the factors *correctness* (violation, correct) and *electrodes* (all 27) showing a significant interaction in the 200–500 time window [*F*(1,26) = 3.61, *p* = 0.003, $\eta_{p}^{2}$ = 0.104] and in the 500–1000 time window [*F*(1,26) = 7.78, *p* < 0.001, $\eta_{p}^{2}$ = 0.201]. To determine the distribution of the effect, a topography-oriented analysis was conducted by dividing the electrodes into posterior and anterior to the central cross-line and into left and right to the central cross-line. A 2 (*distribution*: posterior, anterior) × 2 (*correctness*) repeated measures ANOVA confirmed a larger effect over the anterior than the posterior area in the 200–500 ms time window [*distribution* × *correctness* interaction: *F*(1,31) = 14.22, *p* = 0.001, $\eta_{p}^{2}$ = 0.314], as well as a larger effect over the posterior than the anterior area in the 500–1000 ms time window [*distribution* × *correctness*: *F*(1,31) = 27.35, *p* < 0.001, $\eta_{p}^{2}$ = 0.469]. There were no significant interactions between the factor *correctness* and right vs. left *distribution* (*F* < 1), confirming that the effects were not lateralized. Follow-up analyses revealed a significant P600 effect to violations compared to correct sentences across all posterior electrodes [*F*(1,31) = 11.11, *p* = 0.002, $\eta_{p}^{2}$ = 0.264] but not across all anterior electrodes (*F* < 1). The anterior negativity was significantly larger for violations compared to correct sentences across all anterior electrodes [*F*(1,31) = 5.04, *p* = 0.03, $\eta_{p}^{2}$ = 0.140] but not across all posterior electrodes (*F* < 1).

### Speaker-Dependent Agreement

**FIGURE 2:**
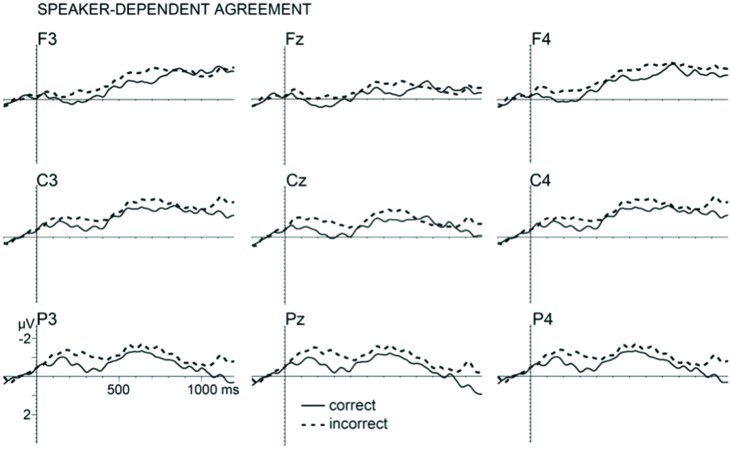
**Grand average ERP’s from nine electrodes elicited by first person incorrect verb agreement (dashed lines) and first person correct verb agreement (solid lines).** Waveforms are filtered (5 Hz high cutoff, 12 dB/oct) for presentation purpose only.

Interestingly, subject–verb-gender agreement violations in the speaker-dependent condition (pragmatic agreement in the first person singular) showed a distinct pattern of results (see **Figure 2**). The lack of variation in effect size across all electrodes was confirmed by a non-significant interaction of the factors *correctness* and *electrodes* in the 100–400 ms time window [*F*(1,26) = 1.14, *p* = 0.29, $\eta_{p}^{2}$ = 0.035], confirming a broadly distributed negativity. There was, however, a main effect of *correctness* [*F*(1,31) = 5.57, *p* = 0.025, $\eta_{p}^{2}$ = 0.152], suggesting that agreement violations resulted in a broadly distributed negativity compared to correct sentences. The topography-oriented analyses showed no significant interactions (all *F*s < 1) and no other significant differences were found in later time windows.

## Discussion

We examined the nature and the time course of the effect of a speaker’s biological gender on subject–verb agreement in spoken Slovak. Despite the large literature on ERP effects observed for gender violations, studies on grammatical agreement features that depend on speaker characteristics extracted from spoken language have been missing. The present study fills this gap by contrasting two different types of gender agreement in Slovak, the speaker-dependent/pragmatic gender agreement and the speaker-independent/syntactic gender agreement. As predicted, disagreement between a formally marked subject and a predicate (e.g., ‘mother-in-law_FEM_ stole_MASC_ plums’) elicited an anterior negativity in the 200–500 ms time window followed by a P600 in the 500–1000 ms time window. The distribution of the anterior negativity was bilateral rather than left lateralised, similar to some previous studies in the auditory modality (e.g., Hahne and Friederici, 2002; Shen et al., 2013), as well as in the visual modality (e.g., Hahne and Jescheniak, 2001; Hagoort et al., 2003; Yamada and Neville, 2007). In line with previous studies on agreement processes, a possible interpretation of this result is that upon hearing the verb, listeners match features between the predicate and the subject nominal phrase leading to the integration of the syntactic and conceptual representations of the utterance. The anterior negativity could be the result of mismatch detection or failed binding between the verbal morphology and the formally marked subject (e.g., Gunter et al., 1997; Hagoort, 2003). The P600 could then indicate a process of reanalysis, revision or recovery from the mismatch detection (e.g., Osterhout and Holcomb, 1992; Hagoort et al., 1993; Friederici, 1995, 2002; Bornkessel-Schlesewsky and Schlesewsky, 2009). Although the present result does not allow resolving the debate on the functional significance of the observed components, it is in line with prior ERP-studies on grammatical gender agreement and extends the electrophysiological evidence on subject–verb-gender agreement to a new language (Slovak).

In contrast to syntactic agreement, incongruencies between the conceptual gender of a speaker and the predicate in the pragmatic condition (e.g., ‘I stole_MASC_ plums’ spoken by a female speaker) resulted in a larger centrally distributed N400-like effect in an early 100–400 ms time window relative to the congruent agreement. The distribution of this effect is comparable to previous studies on speaker integration (Van Berkum et al., 2008; Bornkessel-Schlesewsky et al., 2013) and on subject–verb agreement studies involving person features (e.g., Schirmer et al., 2005; Mancini et al., 2011; Shen et al., 2013). One possible interpretation of this result is that speaker characteristics directly impact the computation of the syntactic relations between words in an utterance but lead to a distinct electrophysiological response than the computation of formal syntactic features, such as the grammatical gender of the subject in the third person. The absence of an anterior negativity could suggest that pragmatic mismatch between the subject and the predicate in the first person may not be treated as a morphosyntactic violation. Similarly, the presence of an early posterior negativity instead of a P600 effect in the pragmatic agreement would suggest that no pure syntactic re-analysis was triggered. This N400-like effect, as well as the rather early onset of this effect, deserves some more discussion.

Previous research has already shown that listeners take into account speaker identity at early stages of meaning construction (Van Berkum et al., 2008; Bornkessel-Schlesewsky et al., 2013). Speaker inconsistency, such as the biologically improbable utterance *I am pregnant* uttered by a male voice elicited a larger N400 than the same sentence uttered in the more probable context of a female voice (Van Berkum et al., 2008). Similarly, false political statements produced by a well-known politician triggered a larger N400 than the same statements produced by a famous news announcer or a control speaker, such as an unknown professor (Bornkessel-Schlesewsky et al., 2013). In contrast, stereotype-driven beliefs about a speaker in self-referent utterances such as *I like to wear lipstick* produced by a man resulted in a larger P600 relative to the same utterance produced by a woman (Lattner and Friederici, 2003). These studies suggest that violations of stereotypical role nouns (e.g., the use of lipstick referring to a man; Lattner and Friederici, 2003) are likely to elicit a P600 (e.g., Osterhout et al., 1997), while semantic-pragmatic violations (as in Van Berkum et al., 2008) are more likely to elicit an N400 (e.g., Irmen et al., 2010). The present study is a valuable addition to this line of research, showing that grammatical agreement processing involving pragmatic aspects (i.e., speaker-related physical/social information) can be modulated in a similar manner, eliciting an N400. Although speaker-related effects have been also observed in syntactic processing (e.g., Hanulíková et al., 2012), they did not involve the type of pragmatic agreement as used in the present study. Syntactic gender errors, such as an incorrect use of determiners in Dutch, lead to a P600 when produced by a native speaker but not when produced by a non-native speaker with a foreign accent, suggesting that the late positivity can be modulated by participants’ inferences about speakers’ linguistic performance. However, the present study is concerned with the pragmatic gender agreement and allows a direct evaluation of the role of speaker identity during processing of sentences that are syntactically correct at the surface level. The results have shown that a speaker’s gender modulates syntactic processing: speaker-based agreement violations elicited a larger N400 compared to speaker-based matching agreement. This suggests that listeners integrate conceptual/semantic information about a speaker during syntactic processing comparable to speaker integration during semantic processing. Since the pragmatic agreement examined in the present study relies on semantic-pragmatic congruity between the conceptual gender of the speaker and the predicate verb, it would be plausible to expect that pragmatic agreement involves the evaluation of speaker characteristics and reflects integration difficulties at the conceptual rather than purely syntactic level.

The question remains, however, how exactly listeners integrate speaker information? Following Nevins et al. (2007), the processing of agreement features could start before hearing the verb in a top–down fashion. Such context-driven top–down processing could be the result of expectation formation. Listeners anticipate certain verbal inflections given a speaker’s gender, which is immediately available for matching agreement features. Indeed, prior research indicates that listeners anticipate linguistic properties (e.g., Connolly and Phillips, 1994; Van Berkum et al., 2005). Van Berkum et al. (2005) has shown that the semantic context of an utterance leads to anticipations of the syntactic gender of a noun and elicits a larger broadly distributed negativity around 300–400 ms for an unpredicted noun relative to a predicted one. Similarly, a word deviating from the expected word in the initial phoneme leads to an early negativity around 300 ms (Connolly and Phillips, 1994). It would therefore be plausible to assume that listeners in the present study anticipated a verbal inflection corresponding to the speaker’s gender and the early N400 may reflect greater speaker-context dependency when such expectations are violated (see also Ye et al., 2006). This would be also in line with suggestions that the degree of semantic-pragmatic predictability is associated with N400 effects (e.g., Kutas and Hillyard, 1984; Van Berkum, 2008).

Listeners could also create similar predictions in the third person singular. However, since the matching between the formal grammatical gender of a nominal phrase and the verbal inflection entails syntactic integration, a P600 instead of an N400 emerges (as in e.g., Nevins et al., 2007). Consequently, the information used by the linguistic system clearly (and perhaps unsurprisingly) differs in the third and first person. Unlike utterances including the third person subject–verb disagreement that is rendered ungrammatical on surface level, the surface structures of the utterances in the pragmatically violated sentences were all grammatically correct. The sentence *lebo som kradla slivky* (‘because I stole_FEM_ plums’) is grammatically incorrect only if spoken by a male speaker. Upon the detection of the incorrect verbal inflection, listeners may trigger a reanalysis of the nominal phrase, but since the personal pronoun is unmarked for gender, no syntactic re-evaluation of the subject takes place. Instead, listeners re-evaluate and revise the speaker information resulting in an N400-like effect, in line with studies on speaker integration during processing of utterance meaning (e.g., Van Berkum et al., 2008; Bornkessel-Schlesewsky et al., 2013), as well as studies on subject–verb agreement involving person feature processing (e.g., Mancini et al., 2011). When the conceptual gender is a salient cue provided by the acoustic signal, the evaluation of the inflectional marker may then be mediated by a pragmatic integration. Since listeners have more time to conceptually and semantically interpret the speaker, the processing of the speaker-dependent verbal inflection could be less syntactically disrupted or the disagreement syntactically less noticeable because the pragmatic feature information decays over time and the sentence *per se* is grammatical (see Deutsch, 1998). The same inflectional marker, however, triggers syntactic integration when the computation is driven by the formally marked grammatical gender of a noun. This interpretation would be in line with proposals according to which the recruitment of non-syntactic information, such as the gender of a speaker, results in N400-like effects, while formally marked syntactic features lead to a structural integration as reflected in a LAN/P600 complex (e.g., Deutsch and Bentin, 2001; Schmitt et al., 2002; Molinaro et al., 2011).

Interestingly, the incongruency effect in the pragmatic agreement arouse very early in time. There are two possible explanations concerning this rather early onset of the negativity. It is plausible to assume that the early onset results from the omnipresent speaker-related information. Semantic-conceptual integration can take place early because gender is present throughout the utterance and listeners might have predictably built expectations about the gender information encoded in the verbal inflectional morpheme (Nevins et al., 2007). Although similar early onsets were observed for semantic integration in the auditory domain (e.g., Holcomb and Neville, 1991; Schirmer et al., 2005; Ye et al., 2006; Bornkessel-Schlesewsky et al., 2013), it should be noted that the time-locking to the inflectional marker of the last syllable of the verb could also contribute to the earlier onset of the effect in the present study. Moreover, the fact that our results do not exactly match the timing of the N400 reported by earlier auditory comprehension work in English (Holcomb and Neville, 1991), Dutch (Hagoort et al., 2003), and German (Friederici et al., 1993), may be due to differences in the investigation of classic semantic violations and pragmatically driven syntactic violations, as well as due to characteristics of the Slovak language. Bornkessel-Schlesewsky et al. (2013) reported an early increased N400 (150–450 ms time-window) to auditory false versus true political statements uttered by a famous politician when compared to the same utterances produced by a famous news announcer or a control speaker (an unknown professor). The result was interpreted in terms of a socially mediated interpretation provided by the speaker identity and suggests that the social status of a speaker influences the neural computation of a linguistic message (Bornkessel-Schlesewsky et al., 2013). In line with their interpretation, the pragmatic N400 in the present study could suggests that listeners combine speaker characteristics and the message (whether semantic or syntactic in nature) very early during linguistic processing in a communicative context.

While the pragmatic agreement computations clearly differ from syntactic agreement, further research is needed to disentangle the possible explanations for the exact nature of the pragmatic agreement compared to the syntactic agreement. It would be interesting to consider further languages with distinct options for social speaker-related grammatical agreement features. Moreover, the future challenge of developing a model of language processing that captures the spread of different types of linguistic information across different languages (e.g., pragmatic vs. syntactic agreement processes) and incorporates both predictive processing and bottom-up feature checking remains. Future studies could also examine individual differences in the computation of pragmatic gender agreement. Specifically, does the gender of the speaker or the gender of the listener and her/his congruency with the speaker changes the sensitivity of detecting pragmatic incongruity between the speaker and the verb participle? And do working memory capacities or empathy (e.g., van den Brink et al., 2012) modulate pragmatic and syntactic agreement computations in different ways?

## Conclusion

Taken together, the results of the present study show that the processing of subject–verb agreement is modulated by the gender of a speaker, and that the integration of speaker and morphosyntax occurs relatively early. Overall, this result extends our knowledge regarding the role of speaker characteristics on the neural correlates of speech processing and is a valuable contribution to cross-linguistic comparisons. Previous research has already shown that listeners integrate a speaker’s identity during meaning construction. The present study has further shown that listeners take the speaker into account during syntactic processing in a similar manner. The linguistic brain thus takes into account all information available to achieve an effortless and successful comprehension of spoken language.

## Conflict of Interest Statement

The authors declare that the research was conducted in the absence of any commercial or financial relationships that could be construed as a potential conflict of interest.
