# Supplementary material for: Electrophysiology of subject-verb agreement mediated by speakers’ gender
Source: Front Psychol. 2015 Sep 15;6:1396. doi: 10.3389/fpsyg.2015.01396 (PMC4569809; doi:10.3389/fpsyg.2015.01396)
Supplement: Supplementary file 1 [file Data_Sheet_1.DOCX]

Appendix: Additional example sentences with English translations. Critical words are underlined. Asterisk indicates an incorrect verbal inflection in a given context.

1^st^ person: pragmatic agreement

1. *Všetci sa smiali, keď som kývol/kývla rukou*.
   ‘Everyone laughed when I waived with the hand.’
2. *Jablká nemali, a tak som siahol/siahla po hruškách.*‘They had no apples and so I reached for pears.’
3. *Hneď keď nasnežilo, tak som vytiahol/vytiahla lyže z pivnice.*
   ‘As soon as snowed, I pulled out skis from the basement.’
4. *V práci tvrdili, že som zašiel/zašla priďaleko.*
   ‘At work they claimed that I went to far.’

3^rd^ person: syntactic agreement

1. *Všetci sa smiali, keď herec kývol/*kývla rukou.*‘Everyone laughed when the actor waived with the hand.’
2. Jablká nemali, a tak zákazníčka *siahol/siahla po hruškách.
   ‘They had no apples and so the customer reached for pears.’
3. *Hneď keď nasnežilo, tak kolega vytiahol/*vytiahla lyže z pivnice.*‘As soon as it snowed, a colleague pulled out skis from the basement.’
4. *V práci tvrdili, že exekútorka *zašiel/zašla priďaleko.*‘At work they claimed that the executor went to far.’
